# Correlation between Orientation Spread and Ear Forming of As-Annealed AA5151 Aluminum Alloy

**DOI:** 10.3390/ma16062408

**Published:** 2023-03-17

**Authors:** Shih-Chieh Hsiao, Chia-Yu Li, Chih-I Chang, Tien-Yu Tseng, Yeong-Tsuen Pan, Jui-Chao Kuo

**Affiliations:** 1 Department of Materials Science and Engineering, National Cheng Kung University, Tainan 70101, Taiwan; 2 Department of New Materials Research and Development, China Steel Corporation, Kaohsiung 81233, Taiwan; 3 ThinTech Materials Technology Co., Ltd., Kaohsiung 82151, Taiwan

**Keywords:** cup drawing, earing, aluminum, texture, analytical model

## Abstract

In the present work, we take the influences of activated slip systems and the orientation spread into account to predict the cup height using analytical earing models and compare the predicted results with experimental results. The effect of boundary conditions of the various stress states and the work hardening exponents are compared and discussed for profile of single crystals. A stress ratio of −0.3 and a hardening exponent of 0.3 are selected for the prediction of earing profiles. The combination of activation of the single slip systems and orientation spread provides the best prediction of deep-drawing profiles. With further consideration of the orientation spread, an increase in the total orientation leads to peak-broadening, i.e., broad and smooth ears. Furthermore, the difference of the height between the maximum and minimum value of cup profiles is reduced because of the orientation spread. The profile for C is found with single ear at 45°, while the other components individually reveal double ears at 35° and 50° for S, at 15° and 45° for B, at 0° and 90° for Cube, at 5° and 90° for r-Cube, and at 15° and 90° for G. Herein, simple analytical earing models are proposed to understand the effects of slip systems and the orientation spread. The deep-drawing profiles are predicted with six major texture components.

## 1. Introduction

Cup drawing is prominently used as an industrial forming process for the manufacturing of beverage cans [1]. The axisymmetric forming of a circular blank into a cylindrical cup, called cup drawing, is the classical type of the sheet forming process known as deep-drawing [2]. Considering the deep drawing of metal sheets, good stretching and bending properties are necessary for all directions to avoid unwanted planar directionality. Thus, good drawability sis the ability of sheet metal to resist thinning at a high plastic strain ratio in axisymmetric forming planar anisotropy, resulting from a preferred crystallographic orientation that contributes to uneven flow [3]. In general, planar anisotropy is undesirable. A cylindrical cup forms different cup heights along the circumference during deep-drawing, which is called earing because of the plastic anisotropy of textured polycrystalline sheets. 

Herein, we will briefly review the definitions of plastic anisotropy and the analytical models of earing. Therefore, Krupkowski and Kawinski [4] and Lankford et al. [5] proposed *K* and *r*-values, respectively, to quantitatively describe the plastic anisotropy. The Lankford parameter of *r*-value called the Lankford value is often used to calculate the ratio of the true width strain $\varepsilon_{w}$ to the true thickness strain $\varepsilon_{t}$ by:

(1) $$r=\frac{\varepsilon_{w}}{\varepsilon_{t}}$$

In addition, the value of the contraction ratio *q* is given by:

(2) $$q=\frac{\varepsilon_{w}}{\varepsilon_{w}+\varepsilon_{t}}=\frac{r}{1+r}$$

where $\varepsilon_{w}$ and $\varepsilon_{t}$ are strains in the width and thickness directions, respectively. Fukui and Kudo [6] (1950) measured the variation of the plastic behavior with directions at 0°, 45°, and 90°, with respect to the rolling direction called Δr-value by:

(3) $$\Delta r=\frac{r_{0}+r_{90}-2r_{45}}{2}$$

where $r_{0}$, $r_{45}$, and $r_{90}$ are the *r*-values at the angle θ of 0°, 45°, and 90° to the rolling direction (RD). Herein, the average *r*-value, denoted by *r_m_*, is calculated by:

(4) $$r_{m}=\frac{r_{0}+r_{90}+2r_{45}}{4}$$

Meanwhile, Hill [7] (1948) proposed a simple quadratic function to calculate the *r*-value for the description of the plastic anisotropy for metal sheets, and the strain ratio of the *r*-value with respect to the angle θ to the rolling direction (RD) is given by:

(5) $$r\left(\theta \right)=\frac{H+\left(2N-F-G-4H\right)sin^{2}\theta con^{2}\theta }{Fsin^{2}\theta +Gcon^{2}\theta }$$

where *F*, *G*, *H*, and *N* are material constants. Barlat et al. [8,9,10,11], Yoon et al. [12,13,14], Inal et al. [2], Zhang et al. [15], and Salehinia and Shahani [16] utilized yield functions to calculate the anisotropy of the yield stress and the strain ratio of the *r*-value following the concept of Hill [7].

Lequeu [17] proposed the percentage of cup height *Z_L_* to measure the plastic anisotropy of sheets by:

(6) $$Z_{L}=\frac{h_{p}-h_{t}}{h_{m}}\times 100\%$$

where *h_p_* and *h_t_* are the average cup height of peaks and troughs, respectively. Meanwhile, the average cup height *h_m_* is the sum of the *h_p_* and *h_t_* values divided by two. Kanetake et al. [18] gave another equation to calculate the percentage of the cup height *Z_K_* by:

(7) $$Z_{K}=\frac{\Delta h}{h_{m}}\times 100\%$$

(8) $$\Delta h=\frac{h_{0}+h_{90}-2h_{45}}{2}$$

(9) $$h_{m}=\frac{h_{0}+h_{90}+2h_{45}}{4}$$

where *h*_0_, *h*_45_, and *h*_90_ are the cup heights at the rotation angle of 0°, 45°, and 90° to the rolling direction, respectively. The delta earing value, Δ*Z*, may be useful, which is defined as:

(10) $$\Delta Z=\frac{{\bar{h}}_{0}+{\bar{h}}_{90}-2{\bar{h}}_{45}}{{\bar{h}}_{0}+{\bar{h}}_{90}}$$

After briefly introducing the measurement of the plastic anisotropy, we come to discuss analytical modeling of earing, because it provides a computationally efficient tool to predict the cup height and minimize earing in industrial manufacturing processes. Assuming that the final cup height consists of a circular base and cylindrical wall, it is, approximately, estimated with the thickness being the same as the initial value reported in Hu et al. in 2002 [19] by:

(11) $$h_{\theta }\approx \frac{R_{p}}{2}\left[{\left(\frac{R_{b}}{R_{p}}\right)}^{2}-1\right]$$

where *R_b_* and *R_p_* are the initial radius of the blank and radius of the punch, respectively.

Based on an early analytical equation developed by Tucker (1961) [20], Lin et al. (1991) [21] calculated the cup height without radial strains, which was further developed by, Li et al. (1997) [22] and Hu et al. (1998) [23], and the final cup height $h_{\theta }$ is composed of $h_{0}$ and $h_{r}$. The average flange elongation is given by:

(12) $${\bar{h}}_{r}=\frac{R_{b}^{2}-R_{p}^{2}}{2R_{p}}+t-h_{0}+0.43r_{p}$$

Meanwhile, the cup height without considering the radial is given by:

(13) $$h_{0}=\frac{1}{4}\left[4\left(R_{b}-R_{p}\right)+\left(4-\pi \right)\left(4r_{p}+t\right)\right]$$

where the radii of the blank and the punch are *R_b_* and *R_p_*, respectively, *r_p_* is the radius of the punch profile, and *t* is the thickness of the blank.

Moreover, Clarke et al. [24] (1994) calculated the cup height at to the rolling direction by:

(14) $$h_{\theta }=t+\left(1-\frac{\pi }{4}\right)\left(2r_{p}+t\right)+\frac{R_{b}^{q+1}-{\left(R_{p}+t\right)}^{q+1}}{\left(q+1\right){\left(R_{p}+\frac{t}{2}\right)}^{q}}$$

where *t*, *R_p_*, *R_b_*, and *r_p_* are the initial thickness of the blank, radius of the punch profile, the initial radius of the blank, and radius of the punch, respectively. In addition, the value *q*, called the contraction coefficient, is equal to the ratio of strains $-\varepsilon_{r}/\varepsilon_{\theta }$.

Yoon et al. (2006) [14] 2006 predicted the total cup height by

(15) $$h_{\theta }=r_{p}+\left(R_{b}-R_{p}\right)+\frac{r_{\theta +90}}{1+r_{\theta +90}}\left(\left(R_{p}-R_{b}\right)+R_{b}\ln\left(\frac{R_{b}}{R_{p}}\right)\right)$$

where the radius of punch and blank are *R_p_* and *R_b_*, respectively, and $r_{\theta +90}=\varepsilon_{r}/\varepsilon_{t}$. The more recent earing models proposed by Yoon et al. (2006 and 2011) [14,25] and Chung et al. (2011) [26] follow the same line; however, they apply a different strain theory.

Baldwin et al. (1945) [27] empirically found that 0/90° earing results from the cube component (100)<001>. Meanwhile, Roberts (1966) [28] observed that 45° earing results from the component of the rolling texture (110)<112> or (113)<211>. Tucker (1961) [20] and Kanetake et al. [18] applied a crystallographic orientation distribution function (ODF) in polycrystalline sheets calculated from texture data to determine the cup height and positions of ears during deep-drawing. Furthermore, Da Costa Viana et al. [29] proposed a model based on the yield locus calculated from ODF to predict the shape of earing by assuming that the radial strain of sheets is inversely proportional to yield stress. Rodrigues and Bate [30] and Van Houtte [31] used yield curves obtained from ODF for the prediction of four ear in aluminum alloys, in which tangential stress is compressive; however, normal stress and radial stress are zero.

Various methods have been developed in the past decades to predict the earing behavior of aluminum alloys during cup deep-drawing with the application of mechanical tests, measurements of texture data, and finite element analysis. Benke et al. [32,33] (2018 and 2020) recently proposed a simple method to calculate the type and magnitude of earing in aluminum sheets, which is based on the texture measurement data of the {200} pole figure. This observation inspires us to understand the correlation between the earing and the {200} pole figure. Thus, in this present study, the anisotropic behavior of earing in AA5151 aluminum alloy sheets was investigated through experimental and simulation approaches to investigate the relationship between the anisotropic behavior of earing and the {200} pole figure.

## 2. Experiments

**Figure 1:**
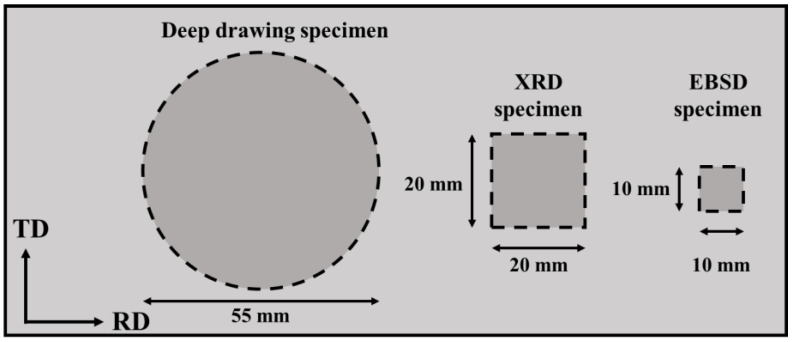
Schematic illustration of the position and the dimension of the examined specimens.

The AA5151 alloy plate provided by China Steel Corporation was used as experiment material to investigate the relationship between the earing behavior and the texture components, and the chemical composition of AA5151 alloy is shown in Table 1. In this study, the AA5151 aluminum alloy sheet was cold rolled to a thickness of 0.25 mm (64.3% reduction), then, subsequently, annealed at 250 °C for 0.5, 1, 2, 3, 4, 7, 10, and 20 h and directly air-cooled to room temperature after annealing. In the first part of experiment, the specimens of AA5151 sheets were prepared for the earing test, texture, and microstructure analysis, and the dimensions of the specimen are given in Figure 1 with RD and TD denoting the rolling direction and transverse direction.

Earing tests were performed on a hydraulic sheet-forming machine by China Steel Corporation. Samples with a radius of 27.5 mm were deformed with a punch having a radius of 16.5 mm and a punch velocity of 0.5 mm s^−1^; the details are presented in Table 2. The cup heights were measured using a laser displacement sensor OMRON ZX2-LD50L0.5M with a precision of 1.5 μm. Then, the cup heights were symmetrized before further analysis. The cup heights at the rotation angle of 0°, 45°, and 90° to the rolling direction were *h*_0_, *h*_45_, and *h*_90_, respectively, which were used to calculate the earing ratio using Equation (7).

The specimens for texture and microstructure analyses were ground using #1500, #2500, and #4000 and cleaned with ethanol. After grinding, they were polished using silica suspensions of particle sizes of 3 μm, 1 μm, 0.5 μm, and 0.25 μm. Finally, electropolishing was conducted using a perchloric acid solution and ethanol at 15 V and −20 °C. The microstructure of the AA5151 alloy was examined using the electron backscatter diffraction (EBSD) technique on the surface parallel to the out-of-plane direction called the ND direction using a Hitachi SU3500 with EDAX HIKARI XP2 at an accelerating voltage of 25 kV under the tilt angle of 70°. The measured area was 200 × 200 ${\mu m}^{2}$ with a step size of 1 μm, and the microstructure was analyzed with the help of the OIM Analysis 8.5 software.

In addition, the Kernel average misorientation (KAM), calculated using the third nearest neighbors, was utilized to characterize the recrystallization in terms of the stored dislocation density. Herein, the value of KAM > 1° was defined as deformed microstructure, whereas that of KAM ≤ 1° was the recrystallized microstructure. Thus, the recrystallization fraction can be quantified by integrating the area fraction in the range of KAM from 0° to 1°.

The texture measurements of AA5151 alloy were examined using X-ray diffraction (XRD) of Bruker D8 advance with Cu$K_{\alpha }$ radiation, having λ of 1.5406 Å on the surface parallel to the out-of-plane direction called the ND direction. Incomplete pole figures of {111}, {200}, and {220} were measured by various tilt angles from 0° to 75° and the rotation angle from 0° to 355° with a constant scanning step of 5°. A defocusing correction was employed by using the measurement of random powder AA5151 alloy. The orientation distribution function (ODF), complete pole figures, and volume fraction of texture components were calculated using the software LaboTex 3.0. Herein, the volume fraction was calculated by integrating the orientation density within a constant volume of $\Delta \varphi_{1}=\Delta \Phi =\Delta \varphi_{2}=10{}^{\circ}$. Texture components used in this work are shown in Table 3.

## 3. Estimation Method of the Cup Height

In the second part of the simulation analysis, we combined both concepts of crystal plasticity and the orientation distribution function (ODF) to estimate the cup height in this study. It was assumed that there was a tensile stress $\sigma_{r}$ in the radial direction, a compressive stress $\sigma_{\theta }$ in the circumferential (or tangential) direction, and a zero stress normal to the sheet surface during a deep drawing in the flange of a blank [20]. Meanwhile, the stress tensor $\sigma_{ij}$ during deep drawing at the flange was expressed as:

(16) $$\sigma_{ij}=\left[\begin{array}{ccc} \sigma_{x} & 0 & 0\\ 0 & \sigma_{y} & 0\\ 0 & 0 & 0 \end{array}\right]$$

and the ratio of radial stress $\sigma_{x}$ to the tangential stress $\sigma_{y}$ is defined as stress ratio *q*.

First, the case of single crystals was considered, which indicated only a single orientation $g\left(\varphi_{1},\Phi ,\;\varphi_{2}\right)$ following Bunge’s definition. According to Sachs theory [34], the deformation occurred only on the particular {111}<110> slip system with the highest resolved shear stress, which can be derived in terms of a given stress tensor $\sigma_{ij}$ using the transformation law for the second-rank tensor by:

(17) $$\tau_{mn}=a_{mi}\;a_{nj}\;\sigma_{ij}$$

where the numbers of *i*, *j*, *m*, and *n* represent the coordinate axes being 1 to 3. The operator $a_{ij}$ expresses the cosine of the angle between two vectors in the subscript. By substituting Equation (16) into Equation (17), the resolved shear stress on a slip system with the normal direction of the slip plane $\vec{n}$ and the slip direction $\vec{d}$ can be expressed as:

(18) $$\tau_{nd}=a_{nx}\;a_{dx}\;\sigma_{x}+a_{ny}\;a_{dy}\;\sigma_{y}$$

where $a_{nx}$ and $a_{dx}$ are the cosine of the angle of the radial direction called $\vec{x}$ direction with the normal direction and the slip direction, respectively. In addition, $a_{ny}$ and $a_{dy}$ are that of the circumferential direction called $\vec{y}$ direction with the normal direction and the slip direction, respectively. Herein, the $\vec{x}$ and $\vec{y}$ directions correspond to the rolling and transverse directions, respectively. Considering a given orientation $\left(\varphi_{1},\Phi ,\;\varphi_{2}\right)$ of a crystal, Miller indices of the rolling and transverse direction can be expressed as:

(19) $$\vec{x}=\left(\begin{array}{c} \cos\varphi_{1}\cos\varphi_{2}-\sin\varphi_{1}\sin\varphi_{2}\cos\emptyset \\ -\cos\varphi_{1}\sin\varphi_{2}-\sin\varphi_{1}\cos\varphi_{2}\cos\emptyset \\ \sin\varphi_{1}\sin\emptyset \end{array}\right)$$

(20) $$\vec{y}=\left(\begin{array}{c} \sin\varphi_{1}\cos\varphi_{2}+\cos\varphi_{1}\sin\varphi_{2}\cos\emptyset \\ -\sin\varphi_{1}\sin\varphi_{2}+\cos\varphi_{1}\cos\varphi_{2}\cos\emptyset \\ -\cos\varphi_{1}\sin\emptyset \end{array}\right)$$

The stress–strain relationship follows a power law relation with an *m* hardening exponent as:

(21) $$\tau =G\cdot \gamma^{m}$$

where τ, γ, and *G* are the shear stress, shear strain, and the material constant, respectively.

Therefore, the strain along the radial direction can be expressed in terms of a shear by:

(22) $$\varepsilon_{xx}=a_{xn}a_{xd}\gamma_{nd}$$

A rotation angle θ was introduced, which represented the angle between the new and initial directions of radial stress to obtain the profile of cup height around a circular blank. The initial direction of the radial stress was called the reference axis, and thus the new orientation $g\left(\theta \right)$ after θ rotation can be written with respect to the reference orientation g as:

(23) $$g\left(\theta \right)=R\left(\theta \right)\cdot g$$

where $R\left(\theta \right)$ is the rotation matrix by rotating the θ angle around the normal direction. After this rotation, the new Miller indices of the rolling direction $\vec{x’}$ and transverse direction $\vec{y’}$ can be expressed as:

(24) $$\vec{x}=\left(\begin{array}{c} \cos\varphi_{1}\cos\left(\varphi_{2}+\theta \right)-\sin\varphi_{1}\sin\left(\varphi_{2}+\theta \right)\cos\emptyset \\ -\cos\varphi_{1}\sin\left(\varphi_{2}+\theta \right)-\sin\varphi_{1}\cos\left(\varphi_{2}+\theta \right)\cos\emptyset \\ \sin\varphi_{1}\sin\emptyset \end{array}\right)$$

(25) $$\vec{x}=\left(\begin{array}{c} \sin\varphi_{1}\cos\left(\varphi_{2}+\theta \right)+\cos\varphi_{1}\sin\left(\varphi_{2}+\theta \right)\cos\emptyset \\ -\sin\varphi_{1}\sin\left(\varphi_{2}+\theta \right)+\cos\varphi_{1}\cos\left(\varphi_{2}+\theta \right)\cos\emptyset \\ -\cos\varphi_{1}\sin\emptyset \end{array}\right)$$

We can acquire a new strain along the radial direction by following the above calculation procedure. If rotating the θ angle from 0° to 360°, it is possible to have the cup height profile around a circular blank for any given orientation g, and the cup height is given by:

(26) $$h_{g}\left(\theta \right)=\frac{\bar{H}}{\bar{\varepsilon }}\varepsilon {\left(\theta \right)}_{xx}+r_{PP}+t_{B}$$

where $\bar{\varepsilon }$, $r_{PP}$, $t_{B}$, and $\bar{H}$ represent the average value of the radial strain, radius of punch profile, the thickness of blank, and average cup height, respectively, as a function of the geometry of the deep-drawing by:

(27) $$\bar{H}=\left(r_{B}{}^{2}-r^{2}\right)/\left(r_{P}+r_{D}\right)$$

where $r_{B}$ is the radius of the blank, $r_{P}$ is the radius of the punch, and $r_{D}$ is the radius of the die. The value of r is given by:

(28) $$r^{2}=2{\left(r_{P}+\frac{t_{B}}{2}\right)}^{2}+\left(r_{P}+\frac{t_{B}}{2}\right)\left(r_{P}-r_{PP}\right)\pi +{\left(r_{P}-r_{PP}\right)}^{2}$$

The effects of stress ratio *q* and hardening exponent *m* were discussed and analyzed in Section 4.2 of this study.

## 4. Results and Discussion

### 4.1. Correlation between Earing and Recrystallization Texture

**Figure 2:**
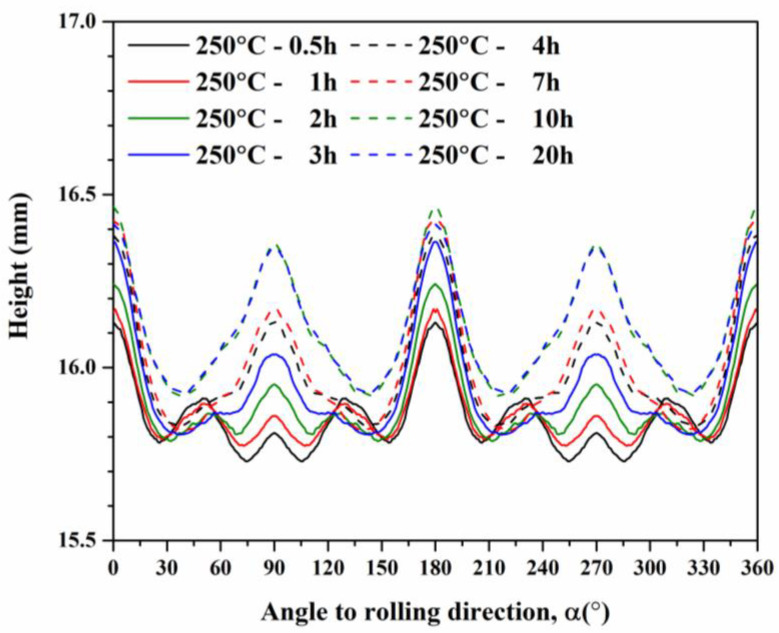
Cup height profiles of deep-drawn AA5151 alloy as a function of the angle calculated from experiment results at 250 °C for 0.5, 1, 2, and 3 h.

**Figure 3:**
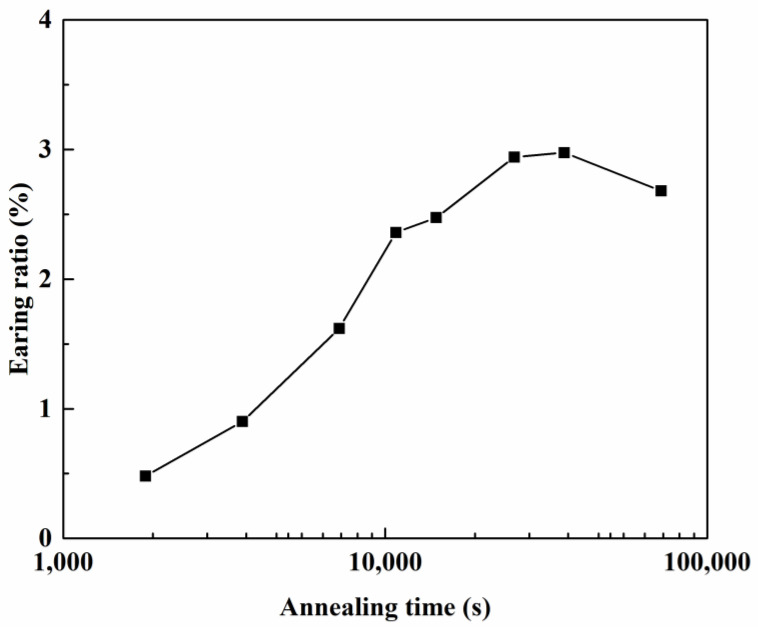
Earing ratios of deep-drawn AA5151 alloy to the annealing time calculated from experiment results.

The cup height profiles in AA5151 alloy are shown in Figure 2 for the annealing time from 0.5 to 20 h. There were eight and four ears from 0.5 to 2 h and from 3 to 20 h, respectively. As the annealing time increased from 0.5 to 20 h, the ears at 0° and 90° increased, whereas the ear at 45° decreased. Benke et al. [33] conducted the deep-drawing experiment of cold rolled Al-Mg alloy and found a sharp ear at 45°. Based on the deep-drawing profiles of single crystals, it can be deduced that the ear at 45° contributed to the deformation texture components, for example, C, S, and B [20]. Figure 2 reveals a decrease of 45° ears and an increase of 0° and 90° ears, which could be deduced to be the contribution of recrystallization texture components, for example, Cube and G. According to Kanetake et al. [18], the percentage of the cup height Z_A_ was calculated by Equation (6), meanwhile, Figure 3 shows the percentage of the cup height with respect to annealing time at 250 °C. Herein, the percentage of the cup height increased as the annealing time increased from 0.5 to 7 h, while it showed a slight decrease after 20 h. Delikanli [35] reported a decrease in ear ratio after annealing.

**Figure 4:**
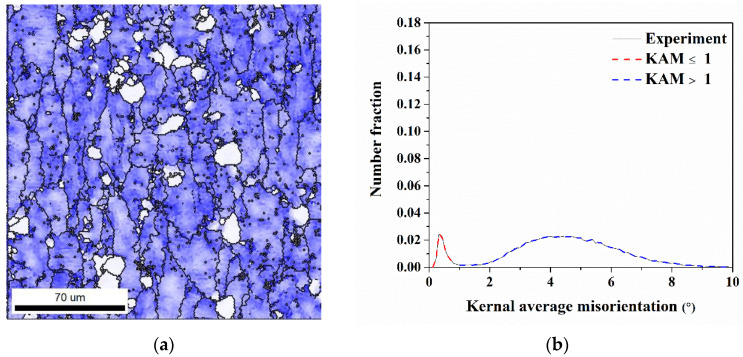

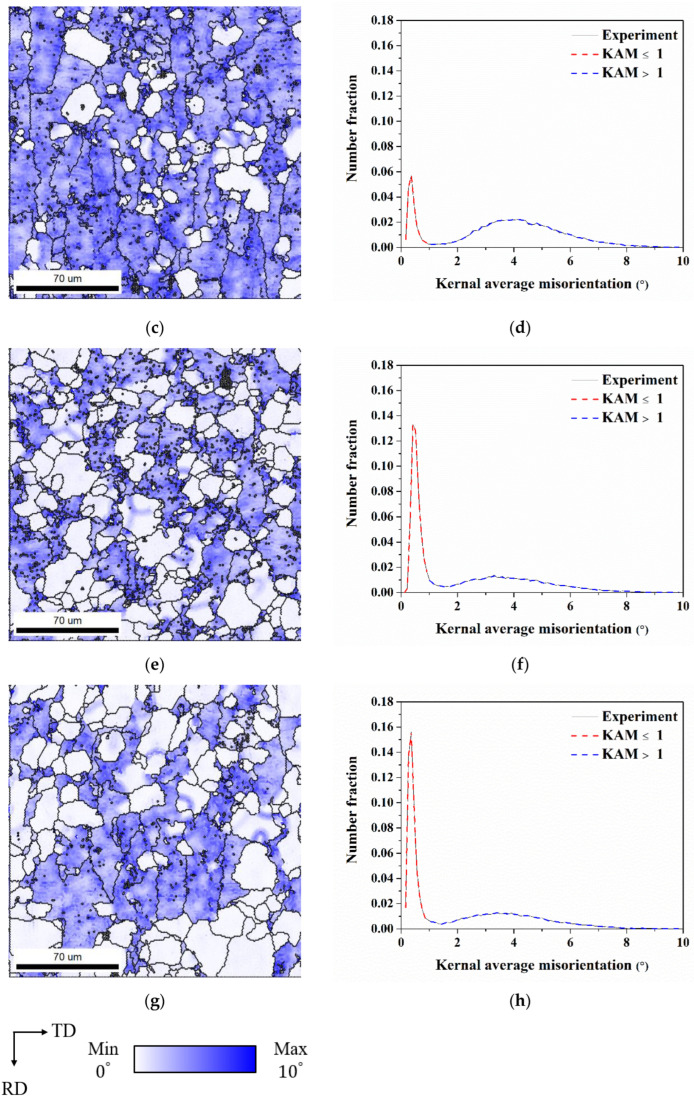
(**a**,**c**,**e**,**g**) KAM maps and (**b**,**d**,**f**,**h**) the corresponding distributions of KAM value in AA5151 alloy at 250 °C for (**a**,**b**) 0.5 h, (**c**,**d**) 1 h, (**e**,**f**) 2 h, and (**g**,**h**) 3 h.

Understanding the relationship between recrystallization and earing behavior is of interest because recrystallization results in the change in microstructure during the annealing process. Herein, the kernel average misorientation technique called KAM was applied to calculate the recrystallization fraction, where the value of KAM indicated the dislocation density of microstructure, which were a large value in deformed microstructure and a small value in the recrystallized microstructure. The KAM maps and their distributions of AA5151 alloy of annealing time from 0.5 to 3 h, for example, are shown in Figure 4. There are two regions, the low and high KAM values represented in white color and blue color, respectively, as shown in Figure 4a,c,e,g. Thus, the recrystallization fraction was quantified by using Gaussian fitting with two peaks as shown in Figure 4b,d,f,h. Moreover, the area with a KAM value < 1° belongs to the recrystallized area, and >1° means a deformed area.

**Figure 5:**
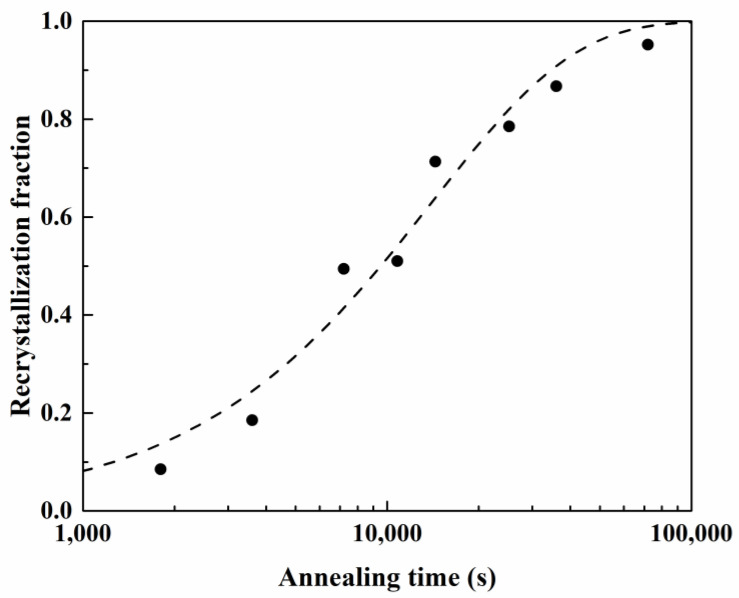
Recrystallization fraction of deep drawn AA5151 alloy to the annealing time calculated from experiment results, where the dashed line is predicted by using the JMAK equation.

For the calculation of the volume fraction of recrystallization, Johnson–Mehl–Avrami–Kolmogorov (JMAK) equation is usually applied by [36]:

(29) $$X=1-exp\left(-kt^{n}\right)$$

where X, t, k, and n denote the recrystallized fraction, time, kinetic parameter, and the JMAK (or Avrami) exponent. The *n* exponent and the *k* value were 0.93 and 1.37 × 10^−4^, respectively, based on the JMAK equation in Figure 5. When both curves of Figure 3 and Figure 5 were directly compared, the recrystallization fraction and the percentage of the cup height were not highly dependent.

**Figure 6:**
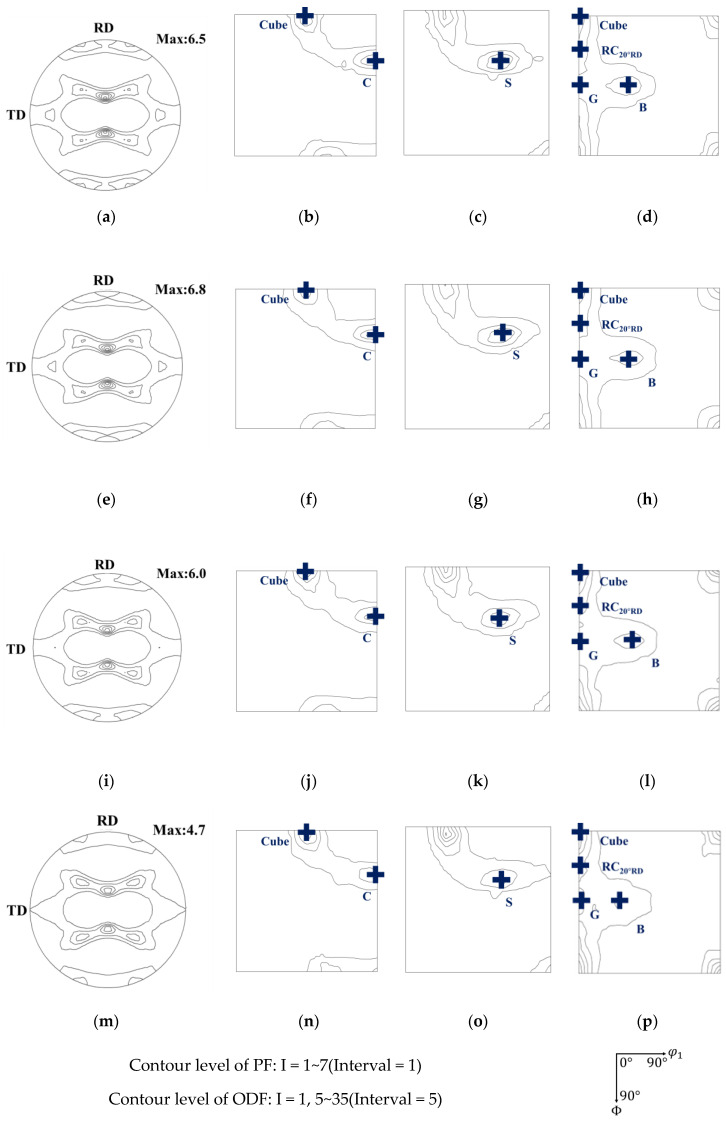
(**a**,**e**,**i**,**m**) {111} Pole figures and $\varphi_{2}$ sections of ODF of at 45°, 65°, and 90° in AA5151 alloy at 250 ℃ for (**a**–**d**) 0.5 h, (**e**–**h**) 1 h, (**i**–**l**) 2 h, and (**m**–**p**) 3 h.

**Figure 7:**
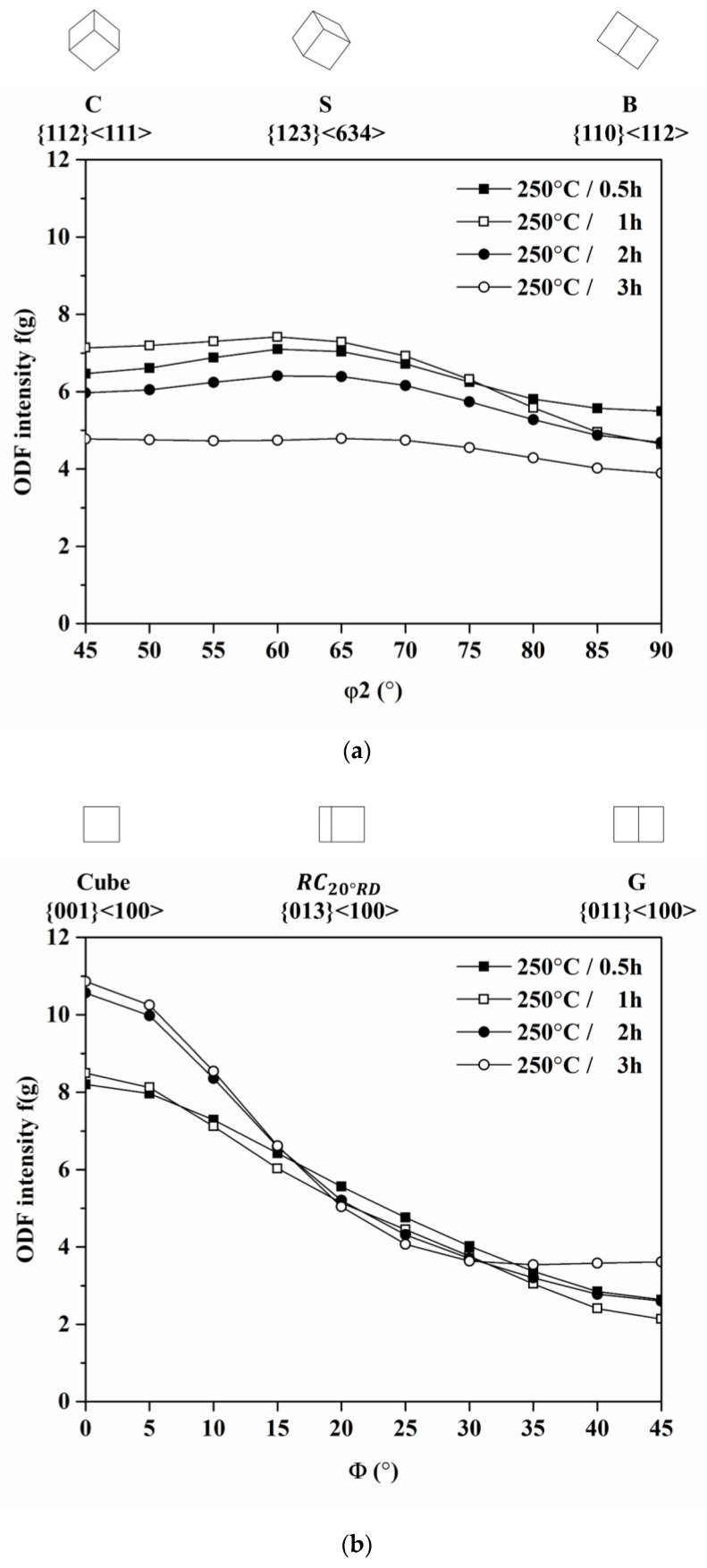
Distribution of orientation intensity along (**a**) β-fiber and (**b**) Cube-Goss fiber of AA5151 alloy at 250 °C for 0.5, 1, 2, and 3 h.

Then, we came to investigate the correlation of texture and earing behavior in the AA5151 alloy. Figure 6 shows {111} pole figures and $\varphi_{2}$ sections of ODF at 45°, 65°, and 90° for the annealed AA5151 alloy, where six major components of C, S, B, Cube, r-Cube, and G and their Miller indices are referred to in Table 3. The r-Cube component means the cube rotated at 20° about the RD direction, and its Miller index was {0 4 11} <1 0 0>. Herein, six major components were found from 0.5 to 3 h of annealing time, as shown in Figure 6. Therefore, the orientation intensity of six components was plotted in β-fiber and Cube-Goss fiber to quantitatively analyze the evolution of each component, as shown in Figure 7. In the case of β-fiber, as the annealing increased, the overall intensity of β-fiber decreased and it became smooth, hence, having a relatively high intensity at C and S. Meanwhile, in the case of Cube-Goss fiber, the overall intensity along this fiber increased as the annealing time increased with the high intensity of the Cube component.

**Figure 8:**
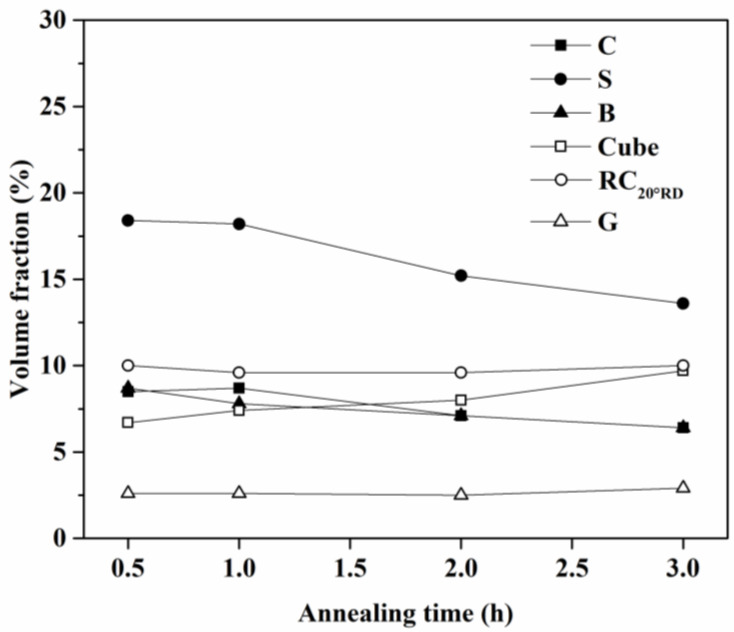
Volume fraction of texture components of AA5151 alloy at 250 °C for 0.5, 1, 2, and 3 h.

The volume fraction of each component was calculated by integrating the intensity around a defined orientation within a misorientation of 10°. Figure 8 and Table 4 show the volume fraction of six components along β-fiber and Cube-Goss fiber. Meanwhile, as the annealing time increased, the volume fractions of C, S, and B components decreased, and the cup height at 45° decreased, as shown in Figure 2. Whereas the Cube, r-Cube, and G components increased, and the cup height at 0° and 90° also increased. This observation coincided with previous studies [37,38]. However, the percentage of the cup height *Z_K_* did not show direct dependence with the volume fraction of six major components. This is because the volume fraction of six major components did not reveal the curve, with respect to the annealing time, as shown in Figure 3. Consequently, we found that the percentage of the cup height *Z_K_* did not show direct dependence with the volume fraction of major components and the recrystallization fraction. This came to a question of the influence of texture components on the formation of ears.

### 4.2. Effect of Activated Slip Systems on the Cup Height

Benke et al. [32] proposed a method to estimate the ear ratio using the {200} pole figures of five major components. This observation indicated a relationship between texture components and cup height. In addition, the formation of the cup height was contributed to the plastic deformation, which can result from the shear by the activated slip systems. Combing both concepts of the Sachs model and orientation distribution function, we calculated the profile of cup height considering a single slip system with respect to one texture component, which is described in Section 4.2. For simplification, the stress ratio and hardening exponent were initially set to a constant value of −1 and +1, respectively. The following discussions of results were limited to the angle interval between 0° and 90° because of the orthotropic symmetry.

**Figure 9:**
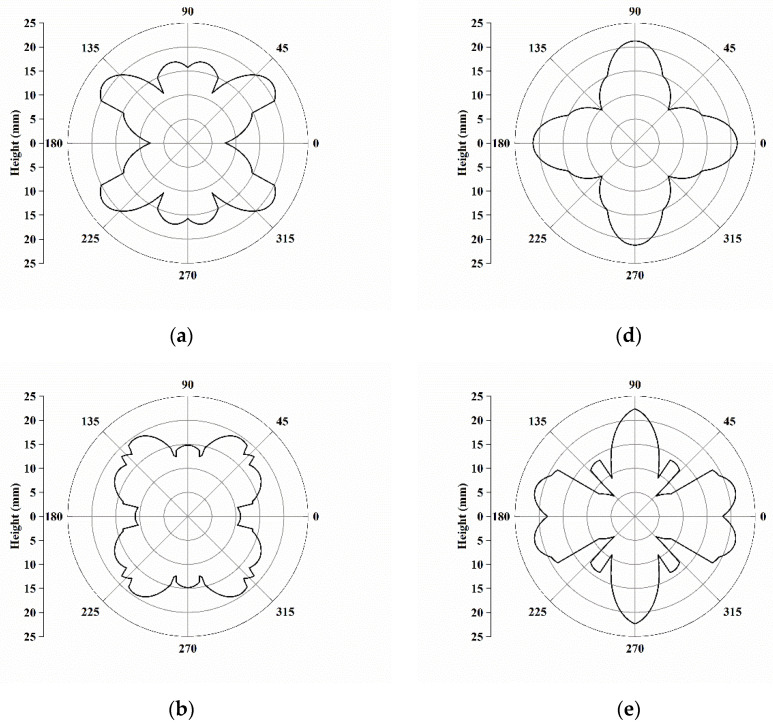

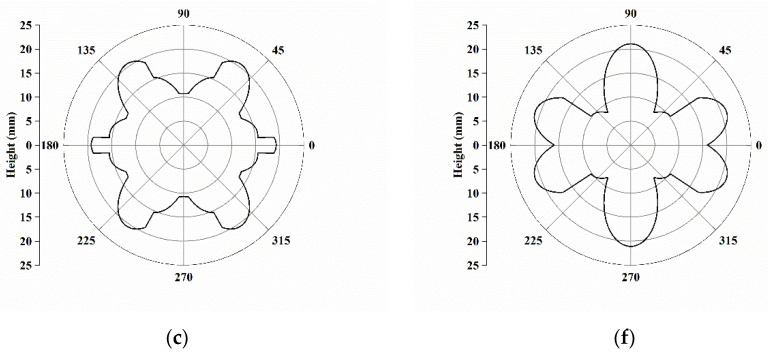
Cup height profiles as a function of the angle for texture components of (**a**) C, (**b**) S, (**c**) B, (**d**) Cube, (**e**) r-Cube, and (**f**) G predicted by assuming stress ratio = −1 and hardening exponent = 1.

Herein, Figure 9 shows the calculated cup heights for C, S, B, Cube, r-Cube, and G, which were plotted along a circle to reveal the symmetric positions of the ear along the 0° and 90° directions, as well as the 45° directions. In general, the contour of the profiles agreed with the experimental deep-drawing profiles of single crystals by Tucker [20]. The ear positions for C, S, and B were at 35°, 55°, and 55°, respectively, which resulted in ears at 45°. Those for Cube, r-Cube, and G were at 0°/90°, 15°/90°, and 19°/90°, respectively, which were very close ears at 0° and 90°. Therefore, we could summarize that the deformation texture components of C, S, and B resulted in the ears at 45°, and recrystallization texture components of Cube, r-Cube, and G contribute to the ears at 0° and 90°.

**Figure 10:**
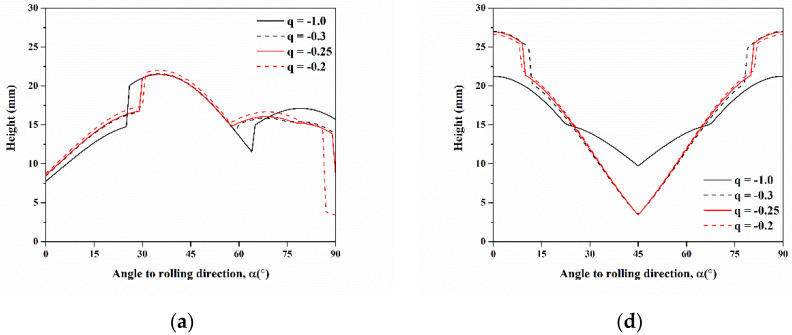

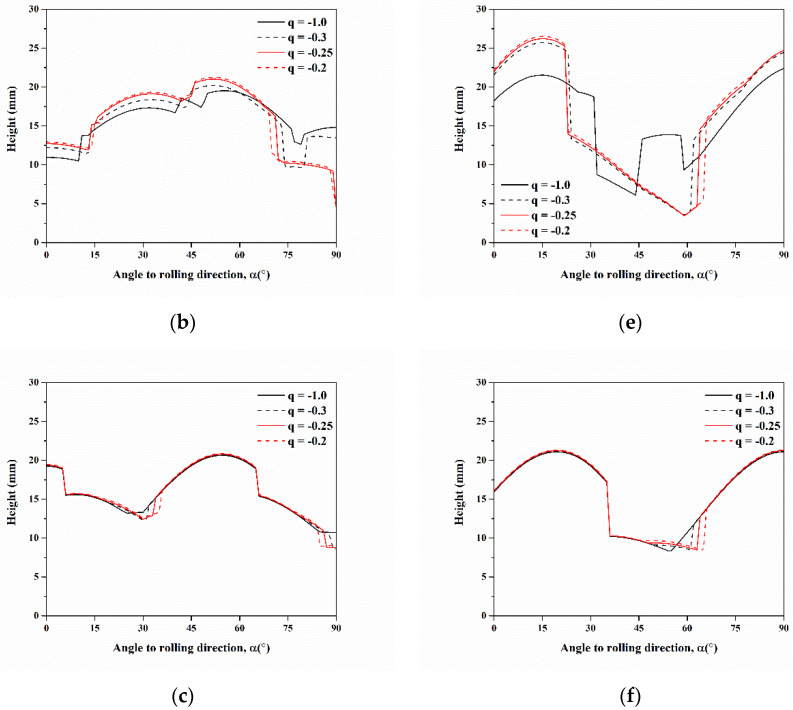
Cup height profiles as a function of the angle for texture components of (**a**) C, (**b**) S, (**c**) B, (**d**) Cube, (**e**) r-Cube, and (**f**) G predicted by assuming constant hardening exponent of 1 and stress ratio q from −0.2 to −1.0.

The cup profile was simulated by varying the value of *q* for six components of C, S, B, Cube, r-Cube, and G to understand the influence of stress ratio *q*, as shown in Figure 10. The cup height for C, S, Cube, and r-Cube components were sensitive to the stress ratio *q*; however, B and G components were insensitive to the stress ratio *q*. As the *q* value increased, it was observed to decrease in height at 90° for C, increase from 15° to 60° and decrease from 70° to 90° for S, increase at 0° and 90° but decrease at 45° for Cube, and increase at 15° and 90° but decrease at 60° for r-Cube. According to Van Houtte et al. [31] and Saimoto et al. [34], the range of stress ratio was between −0.2 to −0.3 in aluminum alloys at the flange region during deep drawing. Compared with the experimental deep-drawn profile, the stress ratio was set to −0.3 because, as the annealing time increased from 0.5 to 20 h, the intensity of ears at 0° and 90° increased, whereas that at 45° decreased.

**Figure 11:**
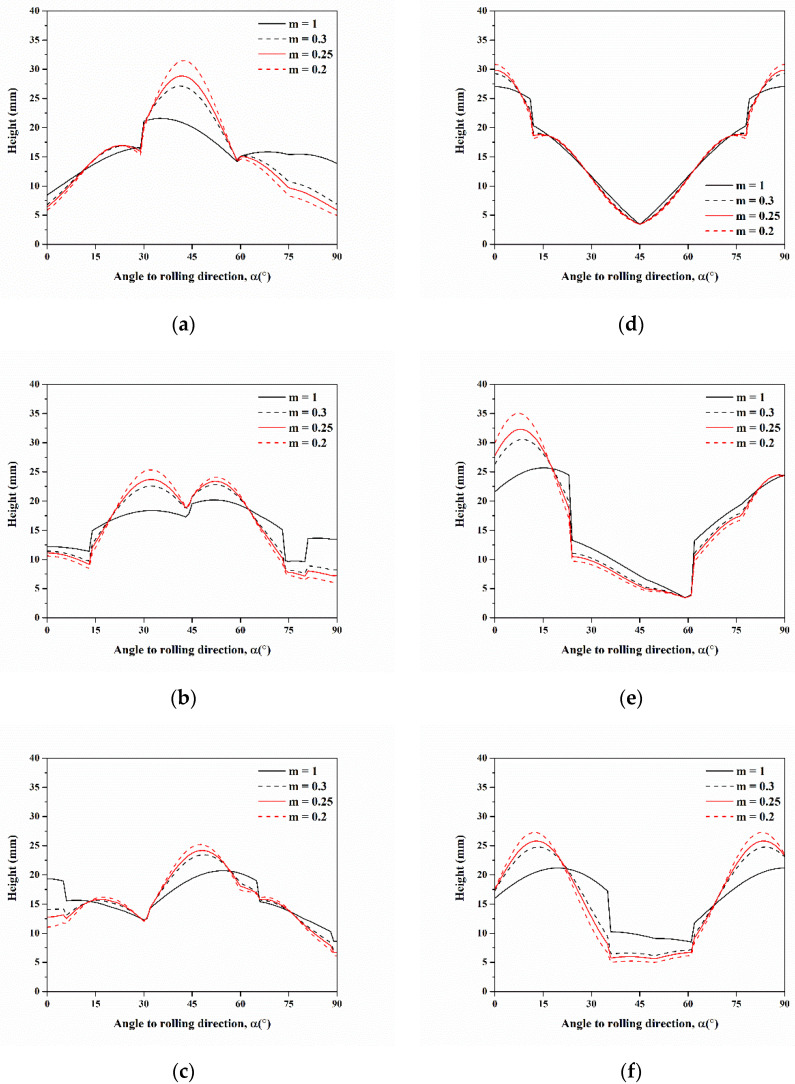
Cup height profiles as a function of the angle for texture components of (**a**) C, (**b**) S, (**c**) B, (**d**) Cube, (**e**) r-Cube, and (**f**) G predicted by assuming hardening exponent m from 0.2 to 1 and stress ratio *q* = −0.3.

**Figure 12:**
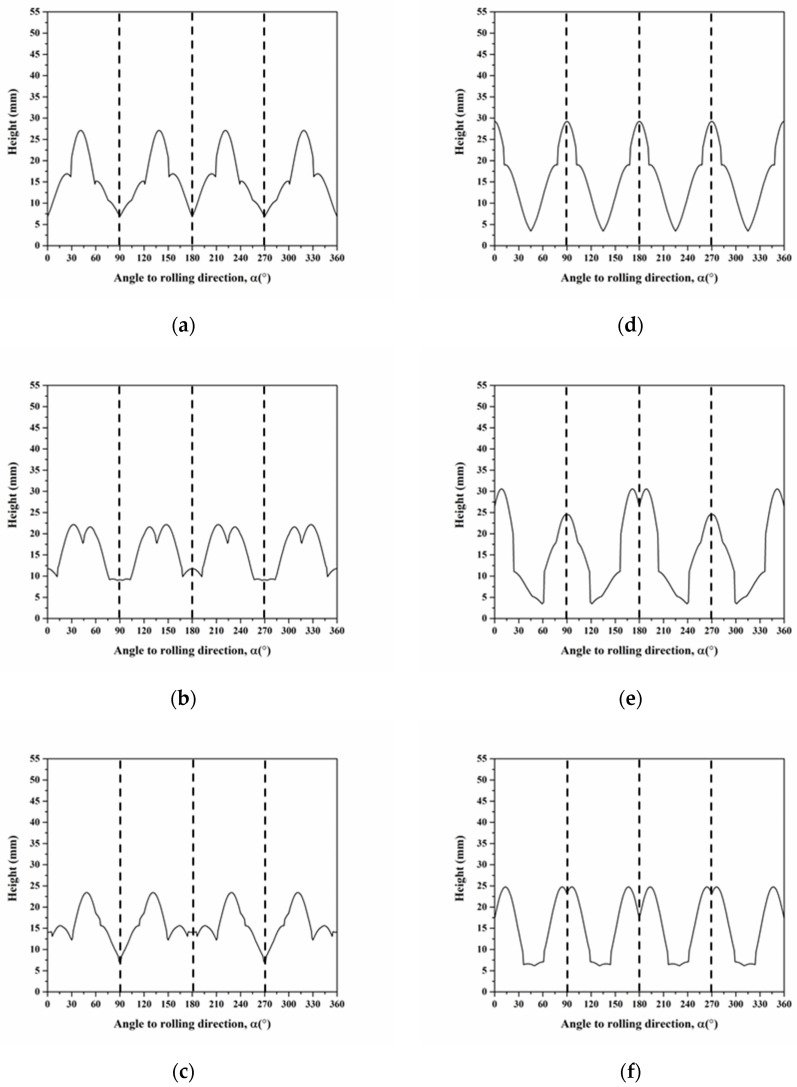
Cup height profiles as a function of the angle for texture components of (**a**) C, (**b**) S, (**c**) B, (**d**) Cube, (**e**) r-Cube, and (**f**) G considering the single slip system.

Then, the profiles of cup height were predicted for six components of C, S, B, Cube, r-Cube, and G under the constant stress ratio of −0.3 by modifying the hardening exponent from 0.2 to 1 in Equation (21) to know the effect of the hardening exponent, as shown in Figure 11. The ears for C, S, and B were at 45°, 30° and 55°, and 45°, respectively. These were at 0° and 90°, 10° and 89°, and 13° and 84° for Cube, r-Cube, and G, respectively. In addition, the formation of clear ears for all six components at m = 0.3 and the intensity of these ears increased with the increasing n value. The strain hardening exponent of Al-Mg alloys was close to 0.3 [39]. Consequently, the m value of 0.3 was selected. After optimizing the stress ratio of −0.3 and the m value of 0.3, the profiles of cup height predicted for C, S, B, Cube, r-Cube, and G are shown in Figure 12. In the range from 0°–90°, the positions of ears for C, S, and B were found at 45°, 30° and 60°, meanwhile, at 50°, these for Cube, r-Cube, and G were at 0° and 90°, at 5° and 90°, and 15° and 90°, respectively. In general, the significant ears for C, S, and B were located at 45°, whereas, Cube, r-Cube, and G were located at 0° and 90°. 

**Figure 13:**
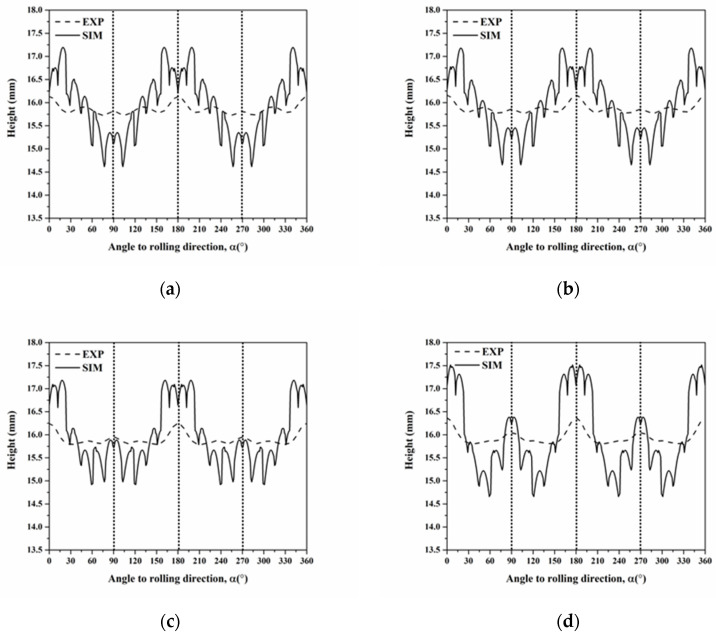
Cup height profiles as a function of the angle in AA5151 alloy at 250 °C for (**a**) 0.5, (**b**) 1, (**c**) 2, and (**d**) 3 h using six components considering the single slip system without orientation spread (Dashed and continuous lines mean experiment and simulation, respectively).

The cup height can be assumed by the contribution of all the texture components in a polycrystalline. Thus, the cup height of $h\left(\theta \right)$ can be summed as the cup height of all texture components g in Equation (23) at the angular position by a counterclockwise angle θ measured to the RD direction. The average cup height of $h\left(\theta \right)$ can be expressed as:

(30) $$h\left(\theta \right)=\frac{1}{n}\overset{n}{\underset{g=1}{\sum }}h_{g}\left(\theta \right)$$

where g and *n* represent the texture component and the number of texture components, respectively. We selected six components of C, S, B, Cube, r-Cube, and G in the annealed AA5151 alloy to calculate the average cup height, based on Equation (30). Moreover, Figure 13 shows the profiles of cup height at 250 °C for 0.5, 1, 2, and 3 h of calculating by using a single slip system. Herein, we discussed two cases of 0.5 and 3 h because the experimental cup height reveals typical four ears and eight ears for both 0.5 and 3. In the case of four ears in Figure 13d, there are six peaks observed at 5°, 17°, 32°, 51°, 65°, and 86°. Furthermore, the cup height continuously decreases from 0° to 60° and increases from 60° to 90°, thereby resulting in two ears at 0° and 90°, i.e., four ears from 0° to 360°. Meanwhile, compared with the peak positions in Figure 12, the 6 peaks observed correspond to the peak positions of G, C, S, C, B, and Cube/r-Cube. These results indicate that the ear at 0° is contributed to by Cube, r-Cube, and G, at 45° by C, S, and B, and at 90° by Cube and r-Cube. As for the eight ears shown in Figure 13a, there are six peaks at 5°, 19°, 35°, 50°, 63°, and 85°, whose positions are close to these positions for 0.5 h. Here, the cup height continuously decreases from 0° to 90° resulting in one ear at 0°, i.e., two ears from 0° to 360°.

**Figure 14:**
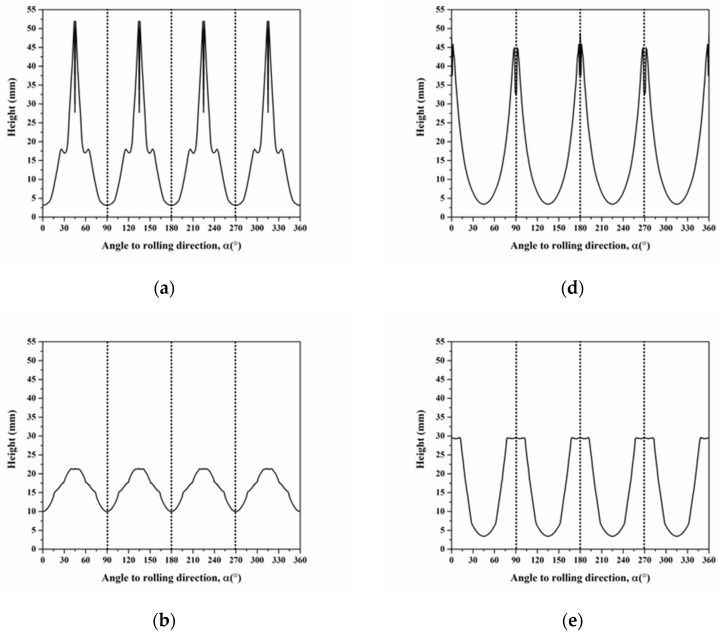

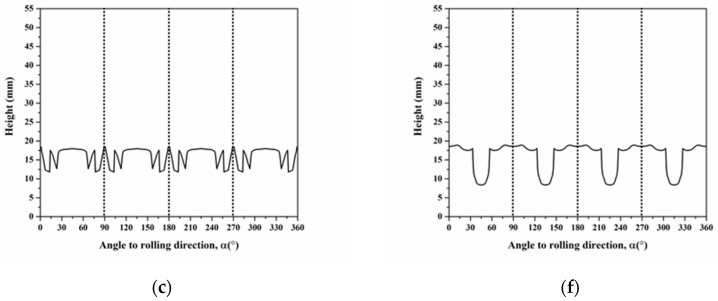
Cup height profiles as a function of the angle for texture components of (**a**) C, (**b**) S, (**c**) B, (**d**) Cube, (**e**) r-Cube, and (**f**) G considering the multi-slip systems.

Considering the activation of multi-slip systems, we adapted the Taylor model with relaxed constraints, called the RC-model [40]. It was adapted to predict multi-slip systems in contrast to a single slip system calculated by the Sachs model in Section 3. In the RC-Taylor model, the relaxed constraint strain was the $\varepsilon_{xx}$ normal strain where the x direction was along the radial direction. The given strain tensor $\varepsilon_{ij}$ with an average circumferential strain of −0.25 was expressed in the macroscopic frame for deep-drawing conditions as the following:

(31) $$\varepsilon_{ij}=\left(\begin{array}{ccc} \varepsilon_{XX} & 0 & 0\\ 0 & -0.25 & 0\\ 0 & 0 & 0.25-\varepsilon_{XX} \end{array}\right)$$

where the $\varepsilon_{XX}$ normal strain is unconstrained. In addition, with the help of the RC model, the profile of cup height can be calculated by the application of the activated slip systems in more than one using Equations (21)–(28). The profiles of cup heights predicted for six components of C, S, B, Cube, r-Cube, and G are shown in Figure 14. In the range from 0° to 90°, the positions of ears for C, S, and B are found at 44°, 45°, and at 1°, 14°, from 32° to 63°, 76°, and 90°. Moreover, these for Cube and r-Cube are at 0° and 90°, while the components of B and G show broaden peaks at 45°, 0°, and 90°, respectively. In general, the significant ears for C, S, and B are located at 45°, whereas for Cube, r-Cube, G, and B are at 0° and 90°.

**Figure 15:**
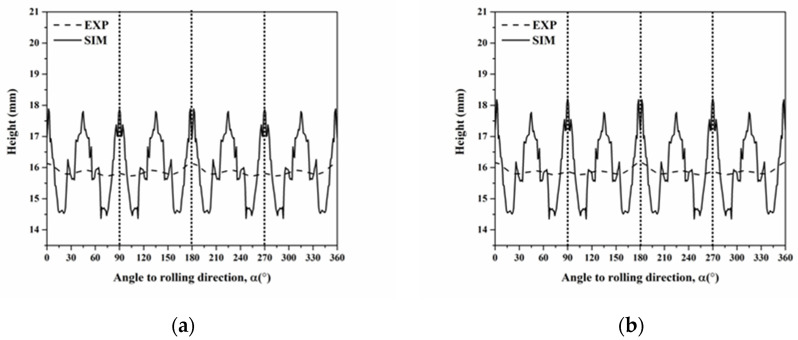

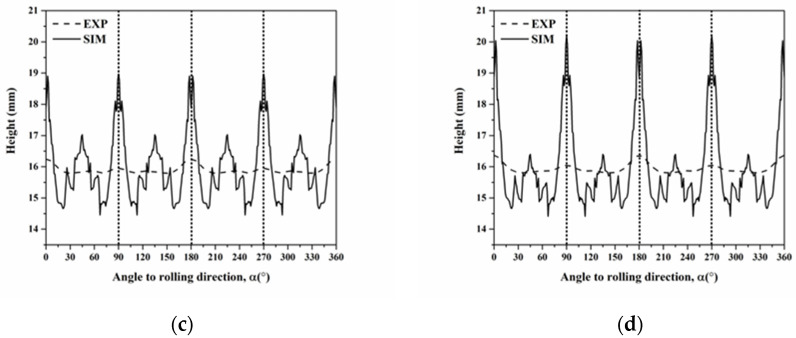
Cup height profiles as a function of the angle in AA5151 alloy at 250 °C for (**a**) 0.5, (**b**) 1, (**c**) 2, and (**d**) 3 h using six components considering the multi-slip systems without orientation spread (Dashed and continuous lines mean experiment and simulation, respectively).

We also selected the six components of C, S, B, Cube, r-Cube, and G in the annealed AA5151 alloy to calculate the average cup height using Equation (30). Figure 15 shows the profiles of cup height at 250 °C for 0.5, 1, 2, and 3 h, calculated using multi-slip systems. Herein, the focus was on two cases of 0.5 and 3 h, respectively. In the case of four ears in Figure 15d, there are five peaks observed at 2°, 26°, 45°, 65°, and 90°, respectively. Meanwhile, compared with the peak positions in Figure 14, these results indicate that the ear at 0° is contributed to by Cube, and r-Cube, at 45° by C and S, and at 90° by Cube and r-Cube. Furthermore, the profile of cup height indicates two major peaks at 0° and 90°, a second peak at 45°, and two third peaks at 30° and 60° close to the second peak at 45°. The profile suggests four ears because the second and third peaks are smaller than the major peaks at 0° and 90°. For the eight ears in Figure 15a, five peaks are found at 2°, 26°, 45°, 65°, and 90°, respectively, whose positions are the same as the positions for 0.5 h in Figure 15d. However, the second peaks are the same as the major peaks at 0° and 90° in magnitude, and the profile shows eight ears. If compared with the profile in Figure 13a using a single slip system, the application of multi-slip systems leads to a sharp ear at 45° in Figure 15a. In addition, the B component contributes to the ears at 0° and 90° aside from the three components of Cube, r-Cube, and G, which are observed considering the single slip system.

### 4.3. Effect of Orientation Spread on the Cup Height

After discussing the influence of the slip-system number, the influence of the orientation spread was then investigated on the profile of cup height with respect to six components. After the deformation of a single crystal, the local lattice rotation within the grain occurred during the straining, thereby resulting in the spread from the ideal orientation. It was often assumed that the deformed orientation distribution indicated a Gaussian distribution with the ideal orientation. Thus, we calculated the orientations that followed a Gaussian function within a given orientation spread from the ideal orientation g to simulate the orientation spread. The spread of orientation can be expressed in terms of a probability function:

(32) $$P\left(\omega \right)=P_{0}exp(-\frac{\omega^{2}}{\omega_{0}{}^{2}})$$

where ω, $\omega_{0}$, and $P_{0}$ are misorientation, half scatter width, and scaling constant at ω=0, respectively. Herein, a total of 50,000 orientations within 10° from the ideal orientation of C, S, B, Cube, r-Cube, and G, respectively, were calculated using the Labotex software to simulate Gaussian distribution.

**Figure 16:**
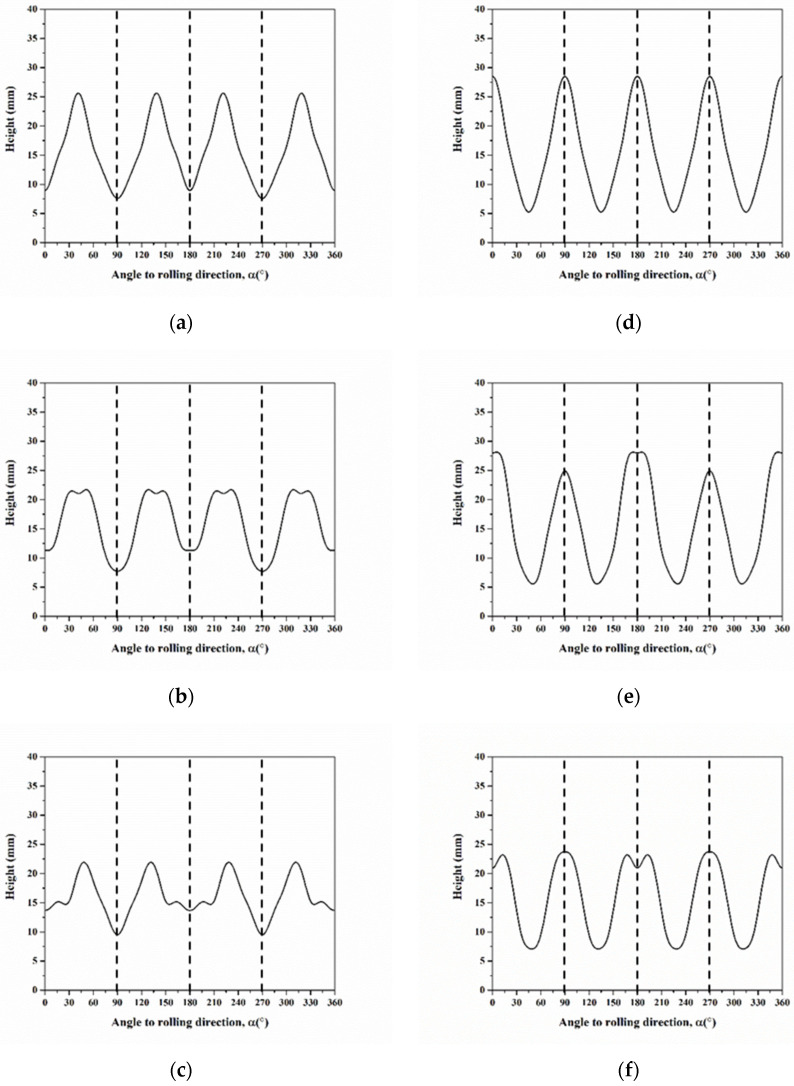
Cup height profiles as a function of the angle for texture components of (**a**) C, (**b**) S, (**c**) B, (**d**) Cube, (**e**) r-Cube, and (**f**) G considering 10° orientation spread.

Figure 16 shows the profiles of cup heights, considering the case of a 10° orientation spread. Herein, the range between 0°–90°, the ears are found at 45° for C, at 35° and 50° for S, at 15° and 45° for B, at 0° and 90° for Cube, at 5° and 90° for r-Cube, and at 15° and 90° for G. In general, the ear at 45° results from the contribution of C, S, and B, whereas that at 0° and 90° result from the contribution of Cube, r-Cube, and G. In addition, considering the single slip system, the profile of cup height shows smoother with the orientation spread, as shown in Figure 16 in comparison with Figure 12.

**Figure 17:**
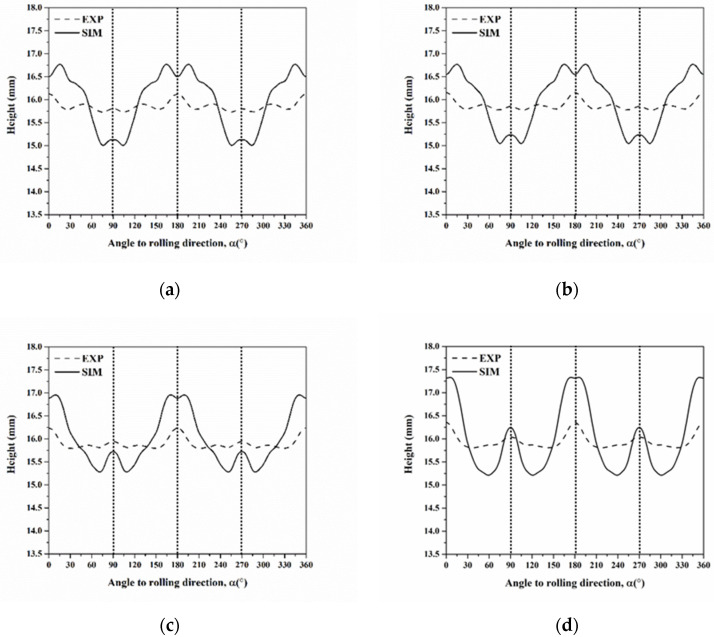
Cup height profiles as a function of the angle in AA5151 alloy at 250 °C for (**a**) 0.5, (**b**) 1, (**c**) 2, and (**d**) 3 h using six components considering the single slip system with orientation spread (Dashed and continuous lines mean experiment and a simulation, respectively).

Based on Equation (30), we also selected six components of C, S, B, Cube, r-Cube, and G in the annealed AA5151 alloy to calculate the average cup height. Furthermore, Figure 17 shows the profiles of cup heights at 250 °C for 0.5, 1, 2, and 3 h. Herein, we discussed two cases of 0.5 and 3 h, respectively. In the case of four ears in Figure 17d, there are two peaks observed at 6° and 90°, and the cup height continuously decreases from 0° to 90°. Comparing with the peak positions in Figure 16, these results indicate that the ear at 6° is contributed to by Cube and r-Cube and 90° by Cube, r-Cube, and G. Meanwhile, in Figure 17a, two peaks are found at 15° and 89° for 0.5 h. The ear at 15° is contributed to by G, and at 89° by Cube, r-Cube, and G. Herein, the cup height continuously decreases from 0° to 90°, thereby resulting in one ear at 0° and a small ear at 90°, i.e., four ears from 0° to 360°. However, it is not observed in the ear at 45°.

In contrast to the application of six orientations using a single slip system without orientation spread in Figure 13, a total of 50,000 weighted orientations were employed to simulate the orientation spread in Figure 17. It suggests that increasing the orientation number leads to peak-broadening, i.e., the formation of broad and smooth ears which is similar to the consideration of the orientation spread.

## 5. Conclusions

In the present work, analytical earing models take the influences of activated slip systems and the orientation spread into account to quantify their predictive capabilities relative to experimental results. Under the assumption of an activated single slip system, the results indicate that the C, S, and B components contribute to the ear at 45°, Cube, r-Cube, and G components to the ear at 0°, and Cube and r-Cube components to the ear at 90°. The assumption of activated multi-slip systems shows that the ear at 45° results from C and S, at 0° and 90° from Cube and r-Cube, respectively. In comparison with the single slip system, the application of multi-slip systems leads to a significant ear at 45°. Considering the orientation spread, it reveals that increasing the orientation number gives rise to peak-broadening, i.e., the formation of broad and smooth ears. Furthermore, the difference between the maximum and minimum cup height values decreases because of the orientation spread. Thus, the boundary conditions of the orientation spread and the application of a single slip system provide the best prediction using the considered analytical earing models.

## Figures and Tables

**Table 1:** Chemical composition of AA5151 aluminum alloy (wt%).

Alloy	Si	Fe	Cu	Mn	Mg	Cr	Zn	Ti	Al
AA5151	0.2	0.35	0.15	0.1	1.50–2.10	0.1	0.15	0.1	Bal.

**Table 2:** The specific dimensions of the tools for cup drawing (mm).

Radius of blank ($r_{B}$)	27.50
Thickness of blank ($t_{B}$)	0.25
Radius of Punch ($r_{P}$)	16.50
Radius of punch profile ($r_{PP}$)	3.18
Radius of die ($r_{D}$)	16.83
Radius of die entry ($r_{E}$)	2.50

**Table 3:** Texture components and corresponding Miller indices in AA5151.

Texture Component	Miller Index
C	{2 2 5}<5 5 4>
S	{1 2 4}<2 1 1>
B	{0 1 1}<5 2 2>
Cube	{0 0 1}<1 0 0>
r-Cube	{0 4 11}<1 0 0>
G	{0 1 1}<1 0 0>

**Table 4:** Volume fraction of texture components (%) and earing ratios of AA5151 alloy at 250 °C for 0.5, 1, 2, and 3 h.

Time (h)	C	S	B	Cube	r-Cube	G	Earing Ratio
0.5	8.5	18.4	8.7	6.7	10	2.6	0.48
1	8.7	18.2	7.8	7.4	9.6	2.6	0.9
2	7.1	15.2	7.1	8	9.6	2.5	1.62
3	6.4	13.6	6.4	9.7	10	2.9	2.36

## Data Availability

Data available on request due to restrictions. The data presented in this study may be available on request from the corresponding author. The data are not publicly available due to the large amount of the data.
